# RDW as A Predictor for No-Reflow Phenomenon in DM Patients with ST-Segment Elevation Myocardial Infarction Undergoing Primary Percutaneous Coronary Intervention

**DOI:** 10.3390/jcm12030807

**Published:** 2023-01-19

**Authors:** Ying Sun, Jian Ren, Li Li, Chunsong Wang, Hengchen Yao

**Affiliations:** 1 Department of Cardiology, Liaocheng People’s Hospital, Shandong University, Jinan 250012, China; 2 Department of Cardiology, Liaocheng People’s Hospital Affiliated to Shandong First Medical University, Liaocheng 252000, China; 3 Department of Cardiology, Liaocheng Dongchangfu People’s Hospital, The Second Affiliated Hospital of Liaocheng University, Liaocheng 252000, China

**Keywords:** no-reflow phenomenon, red blood cell distribution width, diabetes mellitus, acute myocardial infarction

## Abstract

Background: No-reflow phenomenon (NRP) in ST-segment elevation myocardial infarction (STEMI) patients is not infrequent. The predictive value of red blood-cell distribution width (RDW) on NRP has not been explored. Methods: STEMI patients undergoing primary percutaneous coronary intervention (pPCI) were enrolled. Plasma samples were obtained at admission. Participants were divided into two groups according to RDW. Logistic regression and receiver operating characteristic (ROC) curve were performed to evaluate the relationship between RDW and NRP. Subgroup analysis was made between the diabetes mellitus (DM) group and the No-DM group. Results: The high RDW group had a higher NRP compared to the low group. In multivariate logistic regression analysis, DM (adjusted odds ratio [AOR]:1.847; 95% confidence interval [CI]: 1.209–2.822; *p* = 0.005) and hemoglobin (AOR: 0.986; 95% CI: 0.973–0.999; *p* < 0.05), other than RDW, were independent predictors of NRP. RDW (AOR: 2.679; 95% CI: 1.542–4.655; *p* < 0.001) was an independent predictor of NRP in the DM group, but not in the No-DM group. In the DM group, area under the ROC curve value for RDW predicting NRP was 0.707 (77.3% sensitivity, 56.3% specificity (*p* < 0.001)). Conclusions: RDW is a predictor of NRP in DM patients with STEMI, which provides further assistance in clinicians’ decision making.

## 1. Introduction

Cardiovascular diseases, particularly ST-segment elevation myocardial infarction (STEMI), represent a major cause of worldwide mortality. STEMI is caused by complete occlusion of the culprit vessel [1]. According to the guideline recommendations [2], primary percutaneous coronary artery intervention (pPCI) is the most important and rapid way for myocardial reperfusion in STEMI patients. The mortality of STEMI patients has greatly declined because of improved pharmacological treatment and the introduction of pPCI [3]. 

Although the patient undergoing pPCI could gain a successful coronary blood flow restoration, not all of them obtain successful reperfusion [4]. This occurrence is called the no-reflow phenomenon (NRP). The incidence of NRP is approximately 10–30% in STEMI patients [5]. There are many methods to identify NRP, such as a 12-lead surface electrocardiogram (ECG), thrombolysis in myocardial infarction (TIMI)-flow grades, corrected TIMI frame count, myocardial blush grade, cardiac magnetic resonance imaging (CMR), of which CMR is the gold standard for assessment and diagnosis and TIMI-flow grades are the most commonly used for clinical diagnosis [6]. NRP has been proven to be associated with negative ventricular remodeling [6], heart failure (HF) [7], an increased rehospitalization [8] and is an independent predictor of myocardial infarction and death [9,10]. So, there is an urgent need to find biomarkers for the risk stratification and prediction of NRP in STEMI. 

Red blood-cell distribution width (RDW) is a routine parameter of complete blood count analysis, which reflects the volumetric heterogeneity of circulating erythrocytes. RDW is traditionally used in the differential diagnosis of anemia [11]. Studies have proven that RDW can reflect the bone marrow’s response to systemic ongoing inflammation [12,13]. Elevated RDW values are associated with the prognosis of patients with stable angina [14], acute myocardial infarction (AMI) [15], HF [16,17,18] and patients undergoing pPCI [19]. However, whether RDW could be used as a novel biomarker to predict NRP in STEMI patients undergoing pPCI was not yet clear. 

Thus, the objective of this study is to illustrate the predictive value of RDW on the incidence of NRP in patients with STEMI undergoing pPCI; we further explored the predictive ability of RDW for NRP in STEMI patients with diabetes mellitus (DM) and without DM.

## 2. Materials and Methods

### 2.1. Study Population

**Figure 1:**
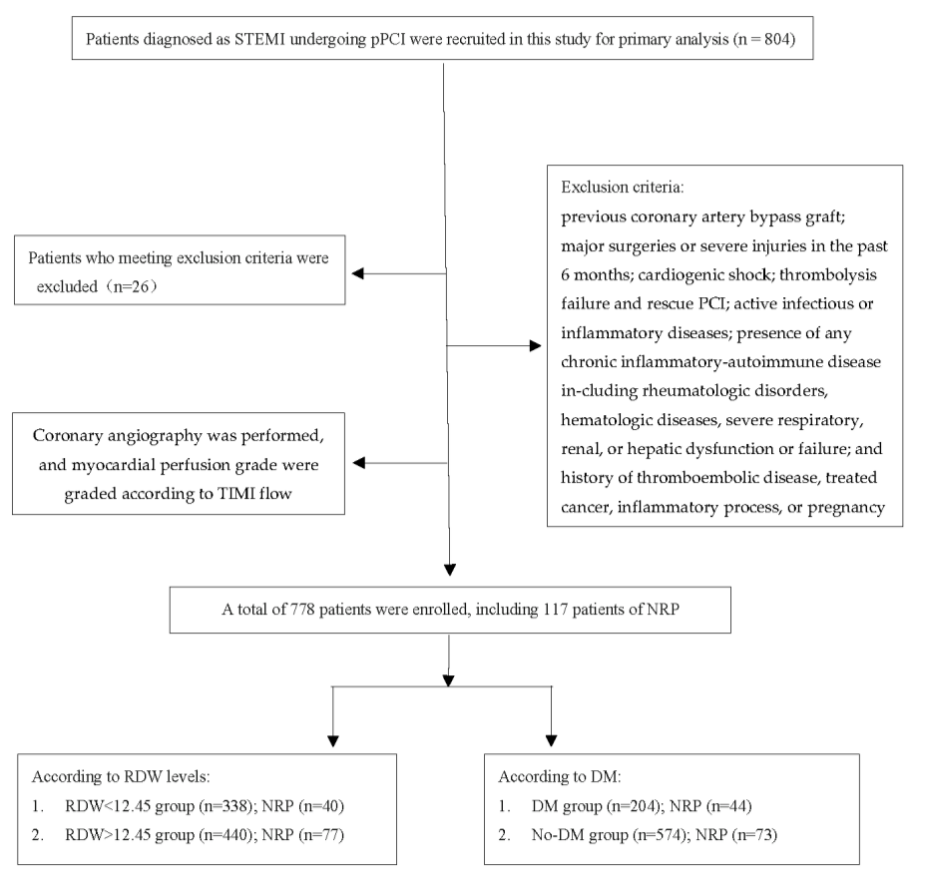
Flow chart illustrating the enrollment and the diagnosis procedure. Abbreviation: STEMI: ST-segment elevation myocardial infarction; pPCI: primary percutaneous coronary intervention; TIMI: thrombolysis in the myocardial infarction; NRP: no-reflow phenomenon; RDW: red blood-cell distribution width; DM: diabetes mellitus.

STEMI patients, admitted into Liaocheng People’s hospital from July 2015 to August 2019, who underwent pPCI within 24 h after the onset of symptoms were recruited in this retrospective study. All the STEMI patients were diagnosed according to the ESC guideline [2]: A chief complaint of continuous typical chest pain for at least 30 min and new persistent ST segment elevation for at least 1 mm in 2 contiguous electrocardiography leads within 12 h of symptom onset or for up to 24 h if there was evidence of persistent ischemia or hemodynamic instability, or new left bundle-branch block in the electrocardiogram, and elevation of cardiac biomarkers, including creatine kinase-MB (CK-MB) and troponin I, above the 99th percentile upper reference limit. The diagnosis was confirmed by coronary angiography in all patients. DM was diagnosed based on plasma glucose criteria, either the fasting plasma glucose (FPG) value ≥ 7.0 mmol/L or the 2-h plasma glucose (2-h PG) value ≥ 11.1 mmol/L during a 75-g oral glucose tolerance test (OGTT) or HbA1C criteria ≥ 6.5% [20]. According to hospital records, baseline characteristics and past medical history including hypertension, diabetes mellitus, smoking status and family history of coronary artery disease (CAD) were collected. Patients with previous coronary artery bypass graft; major surgeries or severe injuries in the past 6 months; cardiogenic shock; thrombolysis failure and rescue PCI; active infectious or inflammatory diseases; presence of any chronic inflammatory-autoimmune disease including rheumatologic disorders, hematologic diseases, severe respiratory, renal, or hepatic dysfunction or failure; and a history of thromboembolic disease, treated cancer, inflammatory process or pregnancy were excluded from our study. The flow chart is shown in Figure 1.

This study was approved by the Medical Ethics Committee of Liaocheng People’s Hospital (NO: 2022035). All procedures were in accordance with the principles of the Helsinki Declaration. 

### 2.2. Blood Sample Collecting and Laboratory Testing

Venous blood samples were obtained from patients by standard venipuncture techniques on admission before the pPCI procedure. Laboratory tests were performed by the emergency laboratory of our hospital. Complete blood counts were performed using the Theisonmicron XN350 according to the manufacturer’s instructions. Biochemical analysis was performed to measure serum total cholesterol (TC), triglyceride (TG), creatinine (Cr), fibrinogen (Fib), D-dimer and blood glucose.

### 2.3. Coronary Angiography and Primary PCI & Assessment of No Reflow Phenomenon

Coronary angiography was performed according to the standard criteria. Epicardial blood flow in the infarct-related artery and myocardial perfusion grade were graded according to the thrombolysis in the myocardial infarction (TIMI) group definitions, a grading system [21,22] to define the scale of coronary blood flow on visual assessment. The coronary blood flow was divided into 4 grades: grade 0, no perfusion; grade 1, penetration without perfusion; grade 2, partial perfusion; grade 3, complete perfusion. NRP was defined as TIMI flow grade ≤ 2 without dissection, stenosis, vasospasm or TIMI flow grade 3 with a TIMI myocardial blush grade < 2, persisting at the end of the PCI procedure [23,24].

### 2.4. Statistical Analysis

Statistical analysis was carried out using SPSS software 23.0 (IBM Corp., Armonk, NY, USA). A Shapiro–Wilk test was used to determine whether continuous data were normally distributed. Normally distributed numerical variables were expressed as mean ± standard deviation, while non-normally distributed data were expressed as median (inter-quartile range). Categorical variables were reported frequency (percentages). The Chi-squared test and Mann–Whitney U test were used for the comparisons of categorical and continuous variables, respectively. Independent factors for predicting the incidence of NRP were calculated by univariate logistic analysis, variables with a *p*-value < 0.2 in univariate analysis were included in multivariate logistic regression models and adjusted odds ratios (AOR) was calculated. A receiver operating characteristic (ROC) analysis was chosen to further explore the applicability of RDW as a potential biomarker in the prediction of NRP. All analyses were two-sided and *p*-values < 0.05 was considered to be statistically significant.

## 3. Results

### 3.1. Patients Characteristics

A total of 778 STEMI patients who underwent pPCI were enrolled in this study, including 574 No-DM patients and 204 DM patients. According to RDW (cut off value) levels, patients were divided into two groups; the results showed that the no-reflow rate in the high-RDW group was significantly higher than that of the low-RDW group (*p* = 0.028). The difference in age, CAD, family history of CAD, hemoglobin, Cr, TC, Fib and D-dimer were also statistically significant (as shown in Table 1). Then, we divided all the patients into two groups according to being with or without DM (as shown in Table 2), the smoking rate, male gender, RDW, Cr, TG and no-reflow rate were significantly different between the two groups. The incidence of NRP is 15.0% (117 of 778 patients) in the whole subjects, 21.6% in the DM group and 12.7% in the No-DM group.

### 3.2. Logistic Regression Analysis for Prediction of NRP

In all subjects, variables *p* < 0.2 in univariate logistic analysis were enrolled into the multiple logistic regression model, the results showed that independent predictors for NRP are DM (AOR: 1.847, 95% confidence interval [CI]: 1.209–2.822, *p* = 0.005) and hemoglobin (AOR: 0.986, 95% CI: 0.973–0.999, *p* = 0.036) but RDW is not a predictor of NRP (as shown in Table 3). Further subgroup analysis was completed, and results showed that in the DM group, RDW is an independent predictor of NRP (AOR: 2.679 (95% CI: 1.542–4.655, *p* = 0.001) (as shown in Table 4). However, RDW is not an independent predictor of NRP in the No-DM group (as shown in Table 5).

### 3.3. ROC Curve to Show the Predictive Value of RDW on NRP

**Figure 2:**
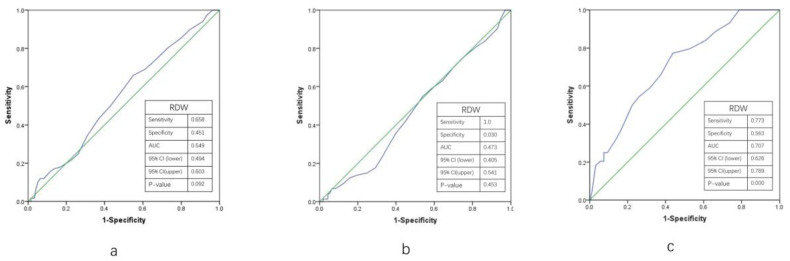
The ROC curve of RDW for predicting no-reflow phenomenon in all patients (**a**), No-DM patients (**b**), and DM patients (**c**). Green lines: diagonal reference line; Blue lines: ROC curve. ROC: receiver-operating characteristics; RDW: red cell distribution width; DM: diabetes mellitus; AUC: area under curve; CI: confidential interval).

The receiver operating characteristic curve (ROC) for RDW in predicting NRP are shown in Figure 2. Results showed that the area under the curve value of RDW for the prediction of NRP in all subjects (a) was 0.549, with 65.8% of sensitivity and 45.1% of specificity (*p* = 0.092), and 0.707 for DM patients (b) (77.3% sensitivity, 56.3% specificity (*p* < 0.001)). However, there were no positive results in the No-DM group (c). 

## 4. Discussion

The main finding in this study is that there is an association between DM and an increased rate of NRP in AMI patients, which is in accordance with prior findings [10]. Moreover, our data uncovered that RDW was associated with the incidence of NRP in DM patients, but not in the No-DM group. As far as we know, this is the first study to elucidate the relationship between RDW and NRP in DM patients.

DM is a disease that causes metabolic disorders, atherosclerosis of blood vessels and microvascular disease. Studies have evaluated the value of DM in predicting the rate of NRP in patients with CAD [25]. In the setting of AMI, accumulating evidence has unearthed that DM can predict the risk of NRP after pPCI [10,26]. Zhao et al. [25] also found that the diabetes duration and higher preoperative blood glucose level were positively associated with the incidence of NRP after PCI. In this study, we confirmed that DM is an independent predictor of NRP, which is in accordance with the former studies. 

Traditionally, RDW has been used as a marker to diagnose the differential of anemia, specifically identifying iron-deficiency anemia [11]. Recently, more and more studies have focused on the influence of RDW on CAD. Studies have shown that RDW levels are associated with the prognosis of patients with stable angina [14], AMI [15], HF [16,17] and patients undergoing pPCI [19]. Further studies have been completed to elucidate the relationship between RDW and NRP. Ghaffari S et al. [27] found that increased RDW was also associated with slow flow in normal coronary arteries. Sahinkus S and his colleagues [28] found that the possibility of developing no-reflow increases 23.48-fold by 1 unit of increase in the level of RDW. A prospective study including one hundred STEMI patients found that increased RDW levels was an independent predictor of NRP after pPCI [29]. In contrast to these findings, the meta-analysis by Zhang E et al. [30] showed no significant association between no-reflow risk and RDW. In our study including 778 STEMI patients, RDW was not an independent predictor of NRP. Therefore, future studies should be made to further confirm the relationship between RDW and NRP in patients with CAD, especially in STEMI patients undergoing pPCI. To date, few studies have investigated the relationship between RDW and NRP in DM patients. To the best of our knowledge, we have, for the first time, confirmed that RDW can be a predictor of NRP in DM other than the No-DM group for patients with AMI. 

Although the pathological and physiological mechanism of NRP has not been fully elucidated, its etiology appears to be multi-factorial. There are several theories that may illustrate the underlying mechanisms of impaired myocardial reperfusion. One is distal microvascular embolism obstruction which is caused by active platelet adhesion and aggregation during the process of plaque rupture [31]. Intracoronary injection of Abciximab improved myocardial perfusion in some studies [32]; additionally, endothelial damage caused by balloon expansion and stent placement can increase endothelin release, the strongest vasoconstrictor, which induces microvascular spasms and increases resistance to the blood flow [33,34]. Another is endothelial dysfunction resulting from prolonged ischemia [35]. Studies have shown that inflammation also plays an important role in the pathophysiological process of NRP [13]. The hyper-coagulation and pro-inflammatory state in diabetic patients, as well as the endothelial dysfunction which is mainly known to be a result of reactive oxygen species (ROS) production and imbalance of endothelium-derived vasodilator and vasoconstrictor mediators in DM [36,37] may well explain this relationship between DM and the incidence of NRP.

Despite these associations, the exact mechanisms underlying the association between RDW and NRP in the DM group remains unclear. Oxidative stress was first found in experimental DM by Matkovics B et al. in 1982 [38], and it has been proven to play an important role in DM and the pathogenesis of diabetic complications. DM is associated with increased oxidative stress resulting from several abnormalities, including hyperglycemia, inflammation and dyslipidemia [39,40]. On the other hand, inflammation inhibits erythrocyte maturation, and accelerates the migration of reticulocytes into the peripheral circulation, thereby increasing RDW [41]. Prior studies had suggested that RDW also plays a role in a number of processes including oxidative stress [42] and inflammation [41]. It is well-documented that the inflammation response plays an important role in the development of AMI. So, we postulate that inflammation may bridge the relationship between a higher RDW and a higher incidence of NRP. Thus, given the uncertainty in the pathways in which RDW acts, further studies with longer study duration and larger samples should be conducted to elucidate these mechanisms. 

## 5. Limitation

There are several limitations in our study. First, all the data of this study came from only one center and a small sample size. Therefore, the results of the present study should be cautiously interpreted. In the future, multi-center studies including a greater sample size may be needed. Second, we only observed the RDW on admission and mechanistic insight was lacking, and further research with dynamic observation to fully understand the mechanism behind the association between RDW and NRP in DM patients who suffered from AMI undergoing pPCI is needed. Studies have shown that the duration of diabetes affects the severity of coronary artery occlusion and the incidence of no-reflow. The diagnosis of DM in this study includes a history of DM and newly diagnosed DM on admission. So, another limitation is that we did not differentiate the duration of diabetes. We hope in the future that subgroup analysis can be conducted to further illustrate the influence of RDW on NRP in DM patients according to the duration of diabetes. Lastly, this is a retrospective study; we hope that further prospective studies should be completed in the future.

## 6. Conclusions

In conclusion, our study showed that DM is an independent predictor of NRP in STEMI patients. For subgroup analysis, we also found that there is a correlation between RDW and NRP in patients with DM and AMI undergoing pPCI, which indicated RDW can be used as a risk-stratification tool for STEMI. 

Although the accurate mechanisms are not determined yet, the way for further investigation of the potential mechanisms of RDW in patients with AMI has been paved. Further studies are needed to unravel the contribution of RDW to the pathogenesis of NRP in patients with AMI.

## Figures and Tables

**Table 1:** Basic characteristics of patients with RDW difference.

Variable	RDW < 12.45	RDW > 12.45	*p*-Value
	n = 338	n = 440	
Age (year)	61 (17)	63 (17)	0.009 *
Smoking, n (%)	184 (54.4)	232 (52.7)	0.635
DM, n (%)	100 (29.6)	104 (23.6)	0.061
Hypertension, n (%)	184 (54.3)	234 (53.2)	0.738
CAD, n (%)	30 (8.8)	60 (13.6)	0.04 *
History, n (%)	32 (9.5)	23 (5.2)	0.022 *
Male, n (%)	256 (75.7)	326 (74.1)	0.6
Time (h)	4.0 (4.13)	4.0 (4.0)	0.748
Heart rate (bpm)	76 (19)	76 (22)	0.179
Hemoglobin (g/dL)	146 (19)	141 (22)	0.000 *
WBC count (×10^9^/L)	9.95 (3.78)	9.66 (4.22)	0.133
NEU (×10^9^/L)	7.61 (3.86)	7.39 (4.05)	0.149
PLT (×10^9^/L)	224 (73)	229 (80)	0.592
LYM (×10^9^/L)	1.34 (1.01)	1.38 (0.94)	0.957
Cr (umol/L)	60.65 (21)	64.40 (20)	0.000 *
TC (mmol/L)	4.79 (1.19)	4.63 (1.36)	0.027 *
TG (mmol/L)	1.47 (1.25)	1.46 (1.21)	0.526
EF (%)	50 (10)	50 (10)	0.967
Fib (ng/mL)	2.92 (0.77)	3.04 (0.89)	0.042 *
D-Dimer (ng/mL)	0.30 (0.42)	0.34 (0.51)	0.032 *
N/L	6.10 (5.83)	5.55 (5.17)	0.281
No-reflow, n (%)	40 (11.8)	77 (17.5)	0.028 *

Abbreviation: RDW: red blood cell distribution width; DM: diabetes mellitus; CAD: coronary artery disease; WBC: white blood cell; NEU: neutrophils; PLT: platelet; LYM: lymphocyte; Cr: creatine; TC: total cholesterol; TG: triglyceride; EF: ejection fraction; Fib: fibrinogen; N/L: neutrophils to lymphocyte ratio; * *p*-values < 0.05.

**Table 2:** Basic characteristics of patients with DM difference.

Variable	No-DM	DM	*p*-Value
	N = 574	N = 204	
Age (year)	62 (18)	61.14 ± 10.81	0.351
Smoking, n (%)	326 (56.8)	90 (44.1)	0.002 *
Hypertension, n (%)	305 (53.1)	113 (55.4)	0.579
Male, n (%)	444 (77.4)	138 (67.6)	0.006 *
Time (h)	4.0 (4.0)	4.0 (4.5)	0.541
Heart rate (bpm)	76 (20)	78.34 ± 15.64	0.200
Hemoglobin (g/dL)	144 (21)	140.36 ± 16.33	0.055
RDW	12.6 (0.8)	12.5 (0.8)	0.043 *
WBC count (×10^9^/L)	9.90 (3.99)	9.75 (4.05)	0.523
PLT (×10^9^/L)	228 (79)	228 (74)	0.772
Cr (umol/L)	64 (20)	60 (21)	0.001 *
TC (mmol/L)	4.70 (1.26)	4.72 (1.51)	0.818
TG (mmol/L)	1.41 (1.14)	1.58 (1.36)	0.001 *
EF (%)	50 (10)	51.6 ± 7.85	0.266
Fib (ng/mL)	2.99 (0.82)	2.98 (0.89)	0.560
D-Dimer (ng/mL)	0.31 (0.44)	0.33 (0.43)	0.813
NEU	7.6 (3.94)	7.32 (4.12)	0.466
LYM	1.35 (0.93)	1.38 (1.06)	0.677
N/L	5.81 (5.24)	5.52 (5.96)	0.417
No-reflow, n (%)	73 (12.7)	44 (21.6)	0.002 *

Abbreviation: DM: diabetes mellitus; RDW: red blood cell distribution width; WBC: white blood cell; PLT: platelet; Cr: creatine; TC: total cholesterol; TG: triglyceride; EF: ejection fraction; Fib: fibrinogen; NEU: neutrophils; LYM: lymphocyte; N/L: neutrophils to lymphocyte ratio; * *p*-values < 0.05.

**Table 3:** Logistic regression analysis of predictors of no-reflow in all subjects.

Variables	OR	95% CI	*p*-Value	Adjusted OR	95% CI	*p*-Value
Age	1.031	1.012–1.050	0.001	1.017	0.997–1.038	0.095
Female gender	1.844	1.213–2.802	0.004	1.268	0.721–2.231	0.409
Smoking	1.412	0.952–2.095	0.086	0.938	0.567–1.552	0.804
Hypertension	1.006	0.678–1.492	0.978			
DM	1.887	1.247–2.856	0.003	1.847	1.209–2.822	0.005 *
Heart rate	0.995	0.983–1.007	0.411			
Hemoglobin	0.979	0.968–0.990	<0.001	0.986	0.973–0.999	0.036 *
WBC count	0.966	0.906–1.031	0.299			
RDW	1.069	0.882–1.295	0.498			
PLT	0.998	0.995–1.001	0.177	0.998	0.995–1.002	0.307
NEU	0.978	0.917–1.043	0.494			
LYM	0.902	0.722–1.126	0.361			
N/L	1.000	0.960–1.042	0.992			
Cr	1.002	0.991–1.013	0.750			
TC	1.012	0.841–1.216	0.903			
TG	0.936	0.802–1.092	0.398			
Fib	1.069	0.843–1.356	0.583			
D-dimer	1.128	0.952–1.337	0.165	1.089	0.908–1.306	0.359
EF	0.988	0.963–1.013	0.347			

Abbreviation: OR: odds ratio; CI: confidential interval; DM: diabetes mellitus; RDW: red blood cell distribution width; PLT: platelet; NEU: neutrophil; LYM: lymphocyte; N/L: neutrophil/lymphocyte; Cr: creatinine; TC: total cholesterol; TG: triglyceride; Fib: fibrinogen; EF: ejection fraction; * *p*-values < 0.05.

**Table 4:** Logistic regression analysis of predictors of no-reflow in the DM population.

Variables	OR	95% CI	*p*-Value	Adjusted OR	95% CI	*p*-Value
Age	1.029	0.996–1.063	0.088	1.015	0.979–1.052	0.429
Female gender	1.426	0.713–2.854	0.316			
Smoking	0.750	0.379–1.484	0.409			
Hypertension	1.077	0.549–2.110	0.830			
Heart rate	1.005	0.984–1.027	0.630			
Hemoglobin	0.982	0.962–1.003	0.094	0.998	0.974–1.022	0.998
WBC count	0.982	0.886–1.088	0.727			
RDW	2.886	1.696–4.913	<0.001	2.679	1.542–4.655	0.001 *
PLT	1.001	0.997–1.006	0.583			
NEU	0.971	0.872–1.080	0.584			
LYM	1.073	0.749–1.537	0.702			
N/L	0.962	0.889–1.041	0.337			
Cr	1.008	0.992–1.024	0.319			
TC	1.021	0.774–1.346	0.886			
TG	0.940	0.766–1.155	0.556			
Fib	1.069	0.734–1.557	0.729			
D-dimer	1.104	0.776–1.569	0.583			
EF	0.967	0.926–1.010	0.133	0.974	0.930–1.019	0.251

Abbreviation: OR: odds ratio; CI: confidential interval; WBC: white blood cell; RDW: red blood cell distribution width; PLT: platelet; NEU: neutrophil; LYM: lymphocyte; N/L: neutrophil/lymphocyte; Cr: creatinine; TC: total cholesterol; TG: triglyceride; Fib: fibrinogen; EF: ejection fraction; * *p*-values < 0.05.

**Table 5:** Logistic regression analysis of predictors of no-reflow in the No-DM population.

Variables	OR	95% CI	*p*-Value	Adjusted OR	95% CI	*p*-Value
Age	1.031	1.008–1.054	0.007	1.011	0.986–1.036	0.392
Female gender	1.964	1.157–3.334	0.012	1.402	0.759–2.589	0.280
Smoking	0.754	0.461–1.233	0.260			
Hypertension	0.952	0.582–1.556	0.843			
Heart rate	0.988	0.972–1.003	0.125	0.993	0.978–1.010	0.425
Hemoglobin	0.978	0.965–0.991	0.001	0.987	0.971–1.003	0.107
WBC count	0.959	0.883–1.041	0.315			
RDW	0.841	0.605–1.170	0.305			
PLT	0.995	0.991–0.999	0.023	0.996	0.992–1.001	0.111
NEU	0.984	0.908–1.067	0.704			
LYM	0.808	0.599–1.090	0.162	0.933	0.686–1.267	0.656
N/L	1.021	0.973–1.072	0.396			
Cr	0.999	0.984–1.014	0.919			
TC	1.004	0.788–1.280	0.974			
TG	0.866	0.684–1.097	0.233			
Fib	1.055	0.776–1.433	0.734			
D-dimer	1.152	0.949–1.400	0.153	1.113	0.903–1.371	0.315
EF	0.996	0.965–1.029	0.829			

Abbreviation: OR: odds ratio; CI: confidential interval; WBC: white blood cell; RDW: red blood cell distribution width; PLT: platelet; NEU: neutrophil; LYM: lymphocyte; N/L: neutrophil/lymphocyte; Cr: creatinine; TC: total cholesterol; TG: triglyceride; Fib: fibrinogen; EF: ejection fraction.

## Data Availability

The data that support the findings of this study are available from the corresponding author upon reasonable request.
